# Changes in feed intake, nutrient digestion, plasma metabolites, and oxidative stress parameters in dairy cows with subacute ruminal acidosis and its regulation with pelleted beet pulp

**DOI:** 10.1186/2049-1891-4-31

**Published:** 2013-08-16

**Authors:** Yongqing Guo, Xiaofeng Xu, Yang Zou, Zhanshan Yang, Shengli Li, Zhijun Cao

**Affiliations:** 1 State Key Laboratory of Animal Nutrition, College of Animal Science & Technology, China Agricultural University, Beijing 100193 China

**Keywords:** Beet pulp, Dairy cow, Nutrient digestion, Oxidative status, Plasma metabolites, Subacute ruminal acidosis

## Abstract

The objectives of this study were to 1) determine the variation of nutrient digestion, plasma metabolites and oxidative stress parameters triggered by induced subacute ruminal acidosis (SARA); and 2) evaluate the ability of pelleted beet pulp (BP) as a replacement for ground corn to alleviate SARA. Eight Holstein-Friesian cows were fed four diets during four successive17-day periods: 1) total mixed ration (TMR) containing 0% finely ground wheat (FGW) (W0); 2) TMR containing 10% FGW (W10); 3) TMR containing 20% FGW (W20); and 4) TMR containing 10% BP as a replacement for 10% ground corn (BP10). The SARA induction protocol reduced the mean ruminal pH from 6.37 to 5.94, and the minimum ruminal pH decreased from 5.99 to 5.41 from baseline to challenge period. Mean ruminal pH increased from 5.94 to 6.05, and minimum daily ruminal pH increased from 5.41 to 5.63, when BP was substituted for corn. The apparent digestibility of nutrients was not affected by the dietary treatments, except that the digestibility of neutral detergent fibre (NDF) and acid detergent fibre (ADF) was reduced in cows fed the W20 diet compared with cows fed the W0 and W10 diets, and cows fed the BP10 diet had higher NDF and ADF digestibility than the cows fed the W20 diet. Cows fed the W20 diet had a lower plasma concentration of β-hydroxybutyrate (BHBA), non-esterified fatty acids (NEFA), cholesterol, triglyceride, and total antioxidative capacity (TAC), and a higher plasma concentration of glucose, insulin, malonaldehyde (MDA), super oxygen dehydrogenises (SOD), and glutathione peroxidase (GSH-Px) than cows fed the W0 diet. Substitution of BP for corn increased concentrations of plasma BHBA and TAC, but decreased concentrations of plasma MDA. Our results indicate that reduction of fibre digestion; the concomitant increase of plasma glucose and insulin; the decrease of plasma BHBA, NEFA, cholesterol, and triglyceride; and changes of plasma oxidative stress parameters are highly related to SARA induced by W20 diets. These variables may be alternative candidates for SARA diagnosis. We also suggest that the substitution of BP for corn could reduce the risk of SARA, increase fibre digestion, and improve the antioxidant status in dairy cows.

## Introduction

Subacute ruminal acidosis (SARA) is one of the most common chronic digestive disorders on intensive dairy farms, and is defined as periods of moderately depressed ruminal pH (the minimum pH varies between 5.2 and 5.6) [1]. Clinical signs of SARA may include reduced dry matter intake (DMI) and milk fat content, rumenitis, laminitis, and liver abscesses, as well as increased death loss [2]. Excessive feeding of nonfibrous carbohydrates (NFC), a rapid increase in levels of dietary NFC, or insufficient rumen buffering are important causes of this metabolic disorder [3].

Measuring rumen fluid pH is the only reliable and accurate tool to diagnose SARA [4]. Because of problems in obtaining samples of rumen fluid, diurnal variation of rumen metabolism, and the lack of pathognomonic signs, SARA is especially difficult to diagnose [5]. Accordingly, there is continued interest in finding quick and simple indicators (e.g., plasma metabolites) as potential diagnostic tools of rumen fermentation pattern and function [6]. Cellulolysis has shown to be impaired by pH below 6.0 to 6.2 [7]; therefore, total tract NDF digestibility might be reduced when rumen pH is lowered. Plasma metabolites were often used to monitor the health and metabolic status of dairy cows, and Ametaj *et al.*[8] indicated that dairy cows fed diets containing high rapidly fermentable carbohydrates could greatly perturb the patterns of plasma metabolites. However, there is limited information on the role of high-grain induced SARA on diurnal perturbations of plasma metabolites in dairy cows. Moreover, it is widely accepted that oxidative stress is positively related to high grain or high starch diets of ruminant animals [9], and that high-producing dairy cows exposed to a high-starch diet are more susceptible to inadequate antioxidant status [10], resulting in a poorly functioning immune system and increased risk of rumenitis as well as laminitis. Therefore, the oxidative stress index could be a general approach used in ruminant medicine in the future [11].

Several dietary strategies proposed for use in preventing SARA, such as sodium bicarbonate and monensin, have been studied [12,13]. However, neither of these methods has consistently maintained higher rumen pH values. Sugar beet pulp (BP) contains approximately 40% neutral detergent fibre (NDF) and is unique in its high content of soluble fibre (especially pectic substances). Soluble fibre fermentation is thought to produce less lactate and propionate than starch fermentation in the rumen, and it does not inhibit cellulose and hemicellulose digestion [14]. Thus, the substitution of corn grain with BP should prevent unfavourable pH decline in the rumen. The goal of this research was to test the hypothesis that SARA induced by increasing the levels of FGW in the diet is associated with alterations in feed intake, nutrient digestion, ruminal pH, selected plasma metabolites, and oxidative stress parameters. Another objective of this study was to evaluate the ability of pelleted BP as a replacement for ground corn to alleviate SARA in the long term.

## Materials and methods

### Experimental design and animal management

Animal care and procedures were approved and conducted under established standards of the College of Animal Science &Technology, China Agricultural University.

Eight multiparous Holstein-Friesian cows (568.5±34.7 kg of BW; 164±15 DIM; mean±SD), four of which were fitted with ruminal cannulas (10 i.d; Bar Diamond, Parma, ID), were fed a series of diets (Table 1) during four successive periods in this experiment: an initial baseline period with 0% finely ground wheat (FGW, geometric mean particle sizes: 600 μm) and starch provided as ground corn, a slowly fermented starch diet (W0 treatment, Baseline period); a second period with 10% FGW, a section of rapidly fermented starch diet (W10 treatment); a third period with 20% FGW, a diet containing a large amount of rapidly fermented starch (W20 treatment, Challenge period); and a final period in which the W20 diet was amended by replacing 10% ground corn with 10% dried, pelleted BP (BP10 treatment). The specific diets were fed sequentially to all eight cows over four 17-d periods (12d adaptation, 5d of measuring), because previous reports suggest that SARA was not immediately and easily reversible [15,16], making other experimental designs (e.g., a Latin square) problematic (e.g., carry-over effects).

**Table 1:** Ingredient composition and chemical analyses of experimental diets

**Item**	**Treatment**^**1**^			
	**W0**	**W10**	**W20**	**BP10**
Ingredient/diets, % of DM				
Corn silage	33.0	27.5	22.0	22.0
Alfalfa hay	15.0	12.5	10.0	10.0
Chinese wild rye	12.0	10.0	8.0	8.0
Corn grain	18.0	18.0	18.0	8.0
Wheat grain	-	10.0	20.0	20.0
Soybean meal	12.6	10.6	9.6	9.6
Cottonseed meal	3.5	3.5	3.5	3.5
Corn distillers grains	1.0	3.0	3.0	3.0
Wheat bran	0.2	-	0.9	1.0
Pelleted beet pulp	-	-	-	10.0
Whole cottonseed	2.0	2.0	2.0	2.0
Mineral-vitamin premix^2^	0.50	0.50	0.50	0.50
Dicalcium phosphate	0.60	0.58	0.47	0.54
Limestone	0.50	0.69	0.92	0.70
Sodium bicarbonate	0.50	0.50	0.50	0.50
Magnesium oxide	0.15	0.15	0.15	0.15
Salt	0.50	0.50	0.50	0.50
Chemical composition, % DM				
CP	15.8	16.2	16.5	16.4
NE_L_, MJ/kg^3^	6.3	6.5	6.7	6.5
NFC^4^	31.9	36.0	42.2	34.8
NDF	41.2	36.2	31.2	38.0
ADF	22.6	19.9	16.1	19.8
fNDF^5^	34.4	28.6	22.9	22.9
Starch	28.5	33.0	36.6	31.0
Ether extract	3.4	3.4	3.3	3.0
Ash	7.7	8.2	6.8	7.8
Ca	0.77	0.77	0.77	0.77
Total P	0.42	0.42	0.42	0.42
F:C^6^	60:40	50:50	40:60	40:60
NFC/NDF	0.77	0.99	1.35	0.92

^1^W0 = TMR containing 0% wheat; W10 = TMR containing 10% wheat; W20 = TMR containing 20% wheat; BP10 = TMR containing 20% wheat plus10% pelleted beet pulp; ^2^Contained (/kg of premix; DM basis): 1,000,000 IU vitamin A; 65,000 IU vitamin D;5000 IU vitamin E; 2,000 mg Fe; 2,550 mg Mn; 5,500 mg Zn; 1,750 mg Cu; 70 mg I; 40 mg Co; 75 mg Se; ^3^Calculated using NE_L_ values of feedstuffs from NRC (2001); ^4^Nonfibre carbohydrates = 100– (NDF% + CP% + Ether extract% + Ash%); ^5^Forage neutral detergent fibre; ^6^Forage to concentrate ratio.

The four diets (Table 1) were formulated to meet or exceed the NRC [3] guidelines for 600 kg multiparous Holstein dairy cows producing 27 kg of milk/d with 4.0% fat. The diets were fed as a total mixed ration (TMR) (CAU-mixer wagon model JZC-200, Beijing, China), and the forage component of the diet was a mixture of corn silage, chopped alfalfa hay, and Chinese wild rye. The moisture content of corn silage was determined weekly and used to adjust the ration. During each data collection period, the particle size distribution of TMR (Table 2) was determined using a Penn State Particle Separator (PSPS) as described by Lammers *et al.*[17].

**Table 2:** **Particle size distribution of the experimental diets (% retained, as-fed basis)**^**2**^

**PSPS sieving**	**Treatments**^**1**^			
	**W0**	**W10**	**W20**	**BP10**
>19 mm	37.5	33.6	31.5	27.0
8 to 19 mm	20.3	18.2	13.7	22.0
1.18 to 8 mm	27.8	30.7	31.1	29.6
<1.18 mm	14.3	16.9	23.2	20.6

^1^W0 = TMR containing 0% wheat, W10 = TMR containing 10% wheat, W20 = TMR containing 20% wheat, BP10 = TMR containing 20% wheat plus10% beet pulp; ^2^Determined using the Penn State Particle Size Separator (PSPS).

The experiment was conducted at the dairy farm of the State Key Laboratory of Animal Nutrition (Beijing, China). The cows were housed in individual tie-stalls bedded with rubber mattresses, and had free access to drinking water throughout the trial. They were fed twice daily, in equal amounts, at 0700 and 1900 h. The diets were fed ad libitum to allow for at least 5–10% orts on an as-fed basis. The cows were milked twice daily at 0630 and 1830 h.

### Sampling and analytical procedures

#### Nutrient intake and digestibility

During d13–d17 of each period, the diets and ort samples of individual cows were harvested daily to calculate nutrient intake. Faecal grab samples (300–500 g fresh basis) were collected on 12 occasions: d14 at 0400, 0900, 1400, 1900 h; d15 at 0500, 1000, 1500, 2000 h; and d16 at 0600, 1100, 1700, and 2200 h. The daily diets, orts, and faecal matter were pooled by dietary treatment and cows, and stored at −20°C until analysis. After the experiment, all the samples were dried at 65°C in a forced-air oven (Model 2000; Experimental Mill, Beijing, China) for 48 h to a constant weight, ground through a 1-mm screen using a Wiley mill (standard model 4; Arthur H. Thomas Co., Philadelphia, PA), and analysed for dry matter (DM), acid detergent fibre (ADF) (method 973.18; AOAC 1990) [18], and starch [19]. The NDF was measured by the method of Van Soest *et al.*[20] with heat-stable α-amylase (A-3306; Sigma Chemical Co., St. Louis, MO), and the sodium sulfite and ash concentration was corrected for the Ankom 200 fibre Analyzer (Ankom Technology, Fairport, NY). The CP was determined by the micro-Kjeldahl method (method 4.2.08; AOAC 1990). Ether extract (method 920.85; AOAC 1990), ash (942.05; AOAC 1990), calcium and phosphorus (method 945.46; AOAC 1990) were also analysed. The chemical composition of the TMR was calculated from the chemical composition of the concentrate mix and the individual forage in the diets. The diets had a similar chemical composition, except for the higher levels of starch and NFC, and the lower levels of NDF in the higher FGW diets.

The acid-insoluble ash (AIA) was used as an intrinsic digestibility marker to estimate nutrient digestibility in the total tract. The AIA in the diets and the faeces were analysed according to Van Keulen and Young [21], using the equation described by Zhong *et al.*[22] to calculate the apparent digestibility of a nutrient in the gastrointestinal tract. The equation is as follows: D = [1– (Ad × Nf)/(Af × Nd)] × 100, where Ad (g/kg) and Af (g/kg) represent the AIA in the diet and faeces, respectively, and Nd (g/kg) and Nf (g/kg) represent the nutrient in the diet and faeces, respectively.

#### Ruminal pH, plasma metabolites and oxidative stress parameters

Ruminal samples were collected for pH analysis. The ruminal fluid (100 mL) was sampled at 0700 (before the meal), 1000, 1300, 1600, and 1900 h at d15 and d16. The samples were collected manually from the anterior dorsal, anterior ventral, medial ventral, posterior dorsal, and posterior ventral locations within the rumen and composited by cow. They were filtered through four layers of cheesecloth. At each sampling time, the pH was measured immediately after collection using a handheld pH electrode (Model pH B-4; Shanghai Chemical, Shanghai, China).

On d17 of each experimental period, 10 mL of blood was collected via tail venipuncture at 0, 3, 6, 9, and 12 h after the morning feeding, into vacutainer tubes (Becton Dickinson, Franklin Lakes, NJ) containing sodium heparin anticoagulant. The plasma was collected after centrifugation at 3,000 × *g* for 10 min, separated into several aliquots, frozen at −20°C, and later analyzed for determination of glucose, insulin, non-esterified fatty acids (NEFA), β-hydroxybutyrate (BHBA), triglycerides, and cholesterol. All plasma-related measurements were analysed in duplicate. The levels of glucose, triglycerides, and cholesterol in plasma were analysed using a clinical auto-Analyzer (Cobas Integra, C701; Hoffmann-La Roche Ltd., Basel, Switzerland). The glucose concentrations were determined using the GOD/PAP test kit (Merit Choice Bioengineering Co., Ltd., Beijing, China). Triglycerides and total cholesterol concentrations were determined following the kit instructions (Shensuo Unf Medical Diagnostic Article Co., Ltd., Shanghai, China), using the enzymatic method. NEFA and BHBA concentrations in plasma were analysed with a Hitachi 7600 automated biochemistry Analyzer (Hitachi Co., Tokyo, Japan). The NEFA concentration was determined using a commercially available kit (Sekisui Medical Co., Ltd., Tokyo, Japan). BHBA dehydrogenase was used for quantifying the plasma concentrations of BHBA using a commercially available kit (Jingyuan Medical Co., Ltd., Shanghai, China). Plasma insulin levels were determined using an insulin radioimmunoassay kit (Beijing North Institute of Biological Technology, Beijing, China) and with a radioimmunoassay system (xh6080; Xi’an Nuclear Instrument Factory, Xi’an, China) according to the manufacturer’s instructions.

The plasma samples collected at 0 and 6 h after the morning feeding were used to test the levels of total antioxidative capacity (TAC), malonaldehyde (MDA) super oxygen dehydrogenises (SOD), glutathione peroxidase (GSH-Px) with colorimetric assay kits (Nanjing Jiancheng Bioengineering Institute, Jiangsu, China) following the manufacturer’s instructions, using a UV visible Recording Spectrophotometer (UV3600, Daojin Corp., Japan). All samples were tested in duplicate. The TAC level was determined by the reaction of phenanthroline and Fe^2+^ using Spectrophotometer at 520 nm [23]. The MDA level was measured by the thiobarbituric acid method [24]. The SOD activity was determined by inhibition of nitroblue tetrazolium reduction due to superoxide anion generation by a xanthine-xanthine oxidase system. The GSH-Px level was determined by using a direct measurement of the remaining GSH after the enzyme-catalysed reaction as described by Hafeman *et al.*[25].

### Statistical analysis

Data on feed intake and digestibility were analysed using the Proc Mixed SAS procedure (SAS Institute, 2002) using the following model: Y_ij_ = μ + T_i_ + C_j_ + ϵ_ij_, where Y_ij_= the dependent variable, μ = the overall mean, T_i_ = the fixed effect of treatment group, C_j_ = the random animal effect, and ϵ_ij_ = the residual error term.

Measurements of ruminal pH, plasma metabolites and oxidative stress parameters were analysed as repeated measures using Proc Mixed SAS procedure (SAS Institute, 2002) using the following model: Y_ijk_ = μ + T_i_ + C_j_ + H_k_ + HT_ik_ + ϵ_ijk_, where Y_ijk_ = the dependent variable; μ = the overall mean; T_i_ = the effect of treatment i; C_j_ = the effect of cow j; H_k_ = the effect of hours post-feeding analysed as repeated measures; HT_ik_ = the interactions between hour k and treatment i, and ϵ_ijk_ = the random residual error. All the above data were compared with Tukey’s range test. Treatment effects were declared significant at *P*≤0.05, and tendencies from *P*>0.05 to ≤0.10. The *P*-values indicated in Tables 2, 3, and 4 refer to the overall diet effects.

**Table 3:** **Effect of dietary treatments on nutrient intake and digestibility in dairy cows**^**1,2**^

**Item**	**Dietary treatment**^**1**^				**SEM**^**3**^	***P*-value**
	**W0**	**W10**	**W20**	**BP10**		
Feed intake, kg/d						
DM	18.87	19.39	19.60	19.40	0.19	0.39
NDF	7.44^a^	6.66^b^	5.77^c^	7.06^ab^	0.14	<0.01
ADF	4.04^a^	3.64^b^	2.93^c^	3.65^b^	0.09	<0.01
CP	3.10^b^	3.25^ab^	3.33^a^	3.31^a^	0.03	<0.01
Ether extract	0.63	0.65	0.63	0.66	0.01	0.07
Starch	5.50^c^	6.66^b^	7.42^a^	6.29^b^	0.13	<0.01
Apparent digestibility of nutrients, %						
DM	72.90	70.54	72.47	70.19	0.62	0.34
NDF	65.96^a^	57.04^bc^	52.33^c^	61.82^ab^	1.36	<0.01
ADF	63.67^a^	55.63^a^	45.57^b^	56.72^a^	1.58	<0.01
CP	75.38	72.72	75.39	71.94	0.62	0.14
Ether extract	70.74	63.04	65.22	65.90	1.30	0.14
Starch	89.38	89.29	89.93	89.35	0.27	0.84

^1^W0 = TMR containing 0% wheat; W10 = TMR containing 10% wheat; W20 = TMR containing 20% wheat; BP10 = TMR containing 20% wheat plus10% beet pulp; ^2^a–c Means with different superscripts in the same row differ (Tukey’s test; *P*<0.05); ^3^SEM = Standard error of the mean.

**Table 4:** **Effect of dietary treatments on ruminal pH and plasma metabolite profiles in dairy cows**^**2,3**^

**Item**	**Dietary treatment**^**1**^				**SEM**^**4**^	***P*-value**		
	**W0**	**W10**	**W20**	**BP10**		**Trt**	**Time**	**Trt × Time**
Ruminal								
pH, Average	6.37^a^	6.01^b^	5.94^b^	6.05^b^	0.05	<0.01	<0.01	0.01
pH, Minimum	5.99^a^	5.63^b^	5.41^c^	5.63^b^	0.07	<0.01	-	-
Plasma								
BHBA^5^, mmol/L	0.82^a^	0.76^ab^	0.68^b^	0.84^a^	0.02	<0.01	<0.01	0.88
NEFA^6^, uEq/L	136.88^a^	123.20^ab^	115.23^b^	111.83^b^	3.37	0.01	<0.01	0.58
Cholesterol, mmol/L	3.71^a^	2.97^b^	2.41^c^	2.55^c^	0.06	<0.01	0.13	0.90
Triglyceride, mmol/L	0.20^a^	0.18^b^	0.17^c^	0.17^bc^	0.01	<0.01	<0.01	0.98
Glucose, mmol/L	3.10^b^	3.13^b^	3.22^a^	3.17^ab^	0.01	<0.01	<0.01	0.97
Insulin, uIU/mL	6.19^b^	7.33^ab^	8.00^a^	7.62^a^	0.22	<0.01	<0.01	0.94

^1^W0 = TMR containing 0% wheat; W10 = TMR containing 10% wheat; W20 = TMR containing 20% wheat; BP10 = TMR containing 20% wheat plus10% beet pulp; ^2^a–c Means with different superscripts in the same row differ (Tukey’s test; *P*<0.05); ^3^Blood samples were taken at 0, 3, 6, 9, and 12 h post morning feeding; ^4^SEM = Standard error of the mean; ^5^β-hydroxybutyrate; ^6^Non esterified fatty acids.

## Results

### Nutrient intake and digestibility

Total DM and ether extract intake were not significantly affected by dietary treatments (Table 3); however, cows fed the W20 diet had a lower NDF and ADF intake (*P*<0.01), and a higher CP and starch intake (*P*<0.01) than the cows fed the W0 diet. The substitution of BP for ground corn increased NDF and ADF intake (*P*<0.01), and decreased starch intake (*P*<0.01). Apparent digestibility of DM, CP, ether extract, and starch was not affected by dietary treatments. However, apparent digestibility of NDF and ADF was lower (*P*<0.01) for cows fed the W20 diet than for cows fed the W0 diet, and cows fed the BP10 diet had a higher (*P*<0.01) NDF and ADF digestibility than the cows fed the W20 diet.

### Ruminal pH, plasma metabolites and oxidative stress parameters

**Figure 1:**
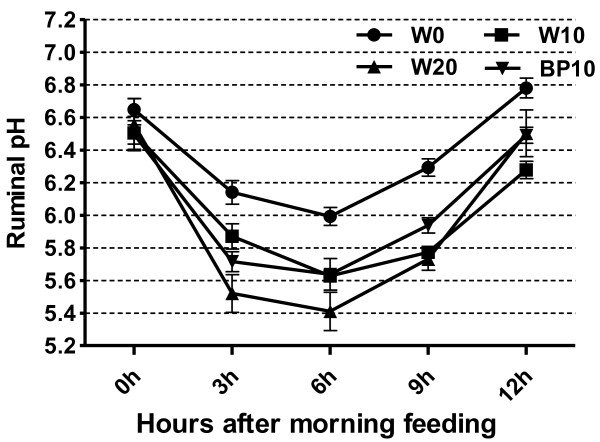
**Effect of dietary treatments on diurnal patterns of ruminal pH in dairy cows.** Data are expressed as mean ± SE.

Ruminal pH profiles on d15 and d16 are illustrated in Table 4 and Figure 1. This SARA induction protocol reduced mean ruminal pH from 6.37 during the baseline period to 5.94 during the challenge period (*P*<0.01); the minimum ruminal pH decreased from baseline to challenge period (5.99 vs. 5.41). Based on the definition of SARA as daily episodes of low rumen pH between 5.2 and 5.6 for at least 180 min/d [1], the most severe rumen pH depression was obtained from cows fed the W20 diet. Ruminal pH from 3 to 9 h after morning feeding was significantly lower (*P*<0.01) for cows fed the W20 diet than for cows fed the other diets. Cows fed the BP10 diet had a higher daily mean ruminal pH (6.05 vs. 5.94) and minimum ruminal pH (5.63 vs. 5.41) than the cows fed the W20 diet. The effects of time and the treatment×time interaction on ruminal pH were significant (*P*<0.05 and *P*=0.01, respectively)” with “on average ruminal pH were significant (*P*<0.01 and *P*=0.01, respectively).

**Figure 2:**
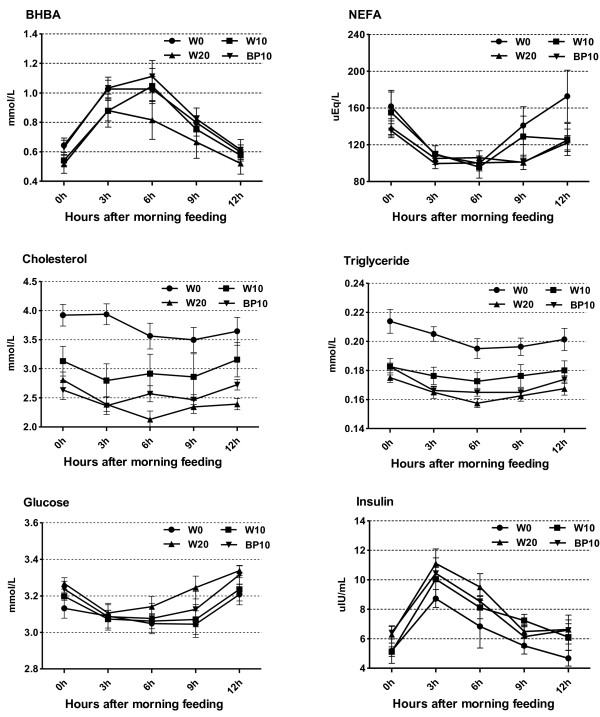
**Effect of dietary treatments on diurnal patterns of plasma metabolites in dairy cows.** Data are expressed as mean ± SE.

Data on diurnal changes of plasma metabolite parameters and oxidative stress parameters are given in Tables 4 and 5, and Figure 2. Compared with the cows fed the W0 diet, cows fed the W20 diet had a lower concentrations of the plasma BHBA (*P*<0.01), NEFA (*P*=0.01), cholesterol (*P*<0.01), triglyceride (*P*<0.01), and TAC (*P*<0.01), and higher concentrations of plasma glucose (*P*<0.01), insulin (*P*<0.01), MDA (*P*<0.01), SOD (*P*<0.01), and GSH-Px (*P*=0.02). The substitution of BP for ground corn increased (*P*<0.01) plasma concentrations of BHBA and TAC, but decreased (*P*<0.01) plasma concentration of MDA. Time after morning feeding had an effect (*P*<0.01) on all plasma metabolite profiles, except the concentration of plasma cholesterol. There was no effect of the treatment×time interaction with respect to all plasma metabolite profiles. The effects of time and the treatment×time interactionon plasma TAC were significant (*P*<0.01). There were no effects of time and the treatment×time interaction with respect to plasma concentrations of MDA, SOD, and GSH-Px, except the concentration of plasma MDA was affected by the treatment×time interaction (*P*<0.01).

**Table 5:** **Effect of dietary treatments on oxidative stress parameters in dairy cows**^**2,3**^

**Item**	**Dietary treatment**^**1**^				**SEM**^**4**^	***P*-value**		
	**W0**	**W10**	**W20**			**BP10**	**Trt**	**Time**	**Trt × Time**
TAC^5^, U/mL	6.14^a^	6.37^a^	5.30^b^	5.94^a^	0.10	<0.01	<0.01	<0.01
MDA^6^, nmol/mL	3.11^c^	3.14^c^	4.39^a^	3.56^b^	0.08	<0.01	0.21	<0.01
SOD^7^, U/mL	65.21^c^	67.63^bc^	73.34^a^	70.88^ab^	0.96	<0.01	0.70	0.07
GSH-Px^8^, U/mL	36.37^b^	41.48^ab^	43.34^a^	41.21^ab^	0.99	0.02	0.06	0.07

^1^W0 = TMR containing 0% wheat; W10= TMR containing 10% wheat; W20 = TMR containing 20% wheat; BP10 = TMR containing 20% wheat plus10% beet pulp; ^2^a–c Means with different superscripts in the same row differ (Tukey’s test; *P*<0.05); ^3^Blood samples were taken at 0 and 6 h post morning feeding; ^4^SEM = Standard error of the mean; ^5^Total antioxidative capacity; ^6^Malonaldehyde; ^7^Super oxygen dehydrogenises; ^8^Glutathione peroxidase.

## Discussion

### Nutrient intake and digestibility

The findings of this study concur with the results of others [4,26]; DMI was not different between the dietary treatments. Paradoxically, reduced DMI was seen as a clinical sign of SARA [27], and several researchers have shown that DMI decreased during induced SARA experiments [28,29]. The effects of induced SARA on DMI were inconsistent, and the causes of this are unclear, probably because of the variance of SARA induction protocol, the severity of SARA, or the individual difference of the cows. Moreover, higher intake and high concentrate included in the W20 diet may have confounded the results. The variation in intakes of CP, NDF, ADF, and starch with changes in the ratio of forage to concentrate (F:C) reflected the chemical composition of the diet consumed because DMI was not affected by F:C.

Here, total tract digestibility of NDF and ADF were reduced in cows fed the W20 diet, although the NDF and ADF intakes were lower than those fed the other diets. This result may be reflected by higher ruminal fermentation of wheat starch of the W20 diet, as well as the negative-associated effects of fibre degradability in the rumen with dietary fermentable carbohydrates [30,31]. Excessively low ruminal pH values can be a problem in dairy cows receiving a diet with large amounts of concentrate or fermentable starch when volatile fatty acid (VFA) production exceeds the buffering ability of the rumen contents. Moreover, fibre digestion can be reduced at pH 6 or less in the rumen [6]. An *in vitro* study by Stensig *et al.*[32] showed that with the higher levels of dietary fermentable starch (wheat), NDF digestion and passage rate were reduced. During the control periods and SARA periods, the 24- and 48-h in situ NDF degradabilities of corn silage were reduced from 44.0 to 37.2% and from 56.1 to 44.8%, respectively [33]. The total tract digestibility of NDF and ADF increased in cows fed the BP10 diet compared with cows fed the W20 diet, and the results might be due to the higher ruminal pH of cows fed the diet inclusion of BP. Our results were consistent with a previous study, which showed that substitution of BP for barley grain in a low-forage diet exhibited a higher apparent digestibility of NDF in dairy cows [34].Wheat grain exhibits faster and more extensive starch and CP degradation in the rumen [35], and it was expected that total tract starch digestibility would be greater in cows fed the higher FGW diets; however, total tract digestibility of CP and starch were not different among treatments. This was unexpected and is difficult to explain.

### Ruminal pH and plasma metabolites

Low ruminal pH was observed in cows fed the W20 diet; the minimum ruminal pH was around 5.4, which was consistent with SARA [1]. SARA induced by increasing the levels of starch and reducing the F:C ratio of the diet results in the accumulation of organic acids (mainly short-chain VFA) in the rumen, as well as reduction in chewing capacity and rumination activity. This in turn leads to an increase in rumen acidity and a decrease in rumen buffering capacity [2]. Increasing ruminal pH with the inclusion of BP in the diet was attributed to the lower production of lactate and propionate from pectic substances in the rumen than the inclusion of corn in the diet [14]. Moreover, compared with the W20 diet, the BP10 diet could increase the levels of NDF and peNDF in dietary DM in our study, and then would increase chewing time and salivary secretion.

The concentrations of plasma BHBA, cholesterol and triglyceride were lower in cows fed the W20 diet throughout the daytime, and the results reveal that SARA had a significant effect on diurnal fluctuations in plasma metabolites. Similarly, a previous study showed that dairy cows fed increasing levels of barley grain were associated with lower concentrations of plasma BHBA and cholesterol [8]. Plasma BHBA comes from the NEFA oxidation in hepatic tissue [36] or from absorbed butyrate [37]. The lower concentration of plasma BHBA is probably due to the variation of its sources and the higher energy density of the W20 diet than that of the other diets. The diurnal fluctuations of plasma BHBA showed the reverse trend compared with those of plasma NEFA in our study, and this is partly explained by the fact that plasma BHBA is produced in the liver and in the rumen epithelium [38]. That is, the higher plasma NEFA concentration in the morning hours could enhance the production of BHBA from NEFA in hepatic tissue, so as to compensate for the relatively low production of BHBA by rumen epithelial cells. The substitution of BP for ground corn increased the concentration of plasma BHBA (mean BHBA concentrations were within the normal range), probably because of the variation of pH value and the fermentation acid concentration in the rumen between the two diets.

Low plasma cholesterol is associated with disturbances of plasma amino acids and severity of the acute phase response [39]. Previous researchers have demonstrated that dairy cows fed large amounts of concentrate or grain-induced SARA often show changes in the composition of rumen microbiota and a rapid accumulation of large amounts of bacterial endotoxin (or lipopolysaccharide, LPS) in the rumen [28,40]. The inverse relationships were found between the concentration of rumen endotoxin and the concentration of plasma cholesterol [6], and low plasma cholesterol levels in cows fed the W20 diets could be related to the systemic inflammatory response triggered by release of endotoxin into the peripheral circulation [28]. Lower plasma triglyceride concentrations were found in cows fed the W20 diet. This is probably due to decreased lipolysis and ruminal biohydrogenation of dietary unsaturated FA at low ruminal pH [41] or higher energy density of the W20 diet, thereby reducing saturated NEFA from the rumen and triglyceride levels in peripheral blood circulation.

The plasma NEFA level has always been used as an indicator of energy status in dairy cows [42]. Cows fed the W20 diet had reduced concentrations of plasma NEFA compared with the other three diets. This finding was similar to the observations of Ametaj *et al.*[8], who demonstrated that the levels of plasma NEFA were lower in cows fed high-grain (barely) diets. The release of NEFA in plasma is based on their mobilization from the adipose triacylglycerol (TG) stores through the process of lipolysis. Increasing the proportion of FGW in the diet could potentially improve the energy balance of dairy cows. Moreover, the abundant availability of ruminal propionate (data not shown) and a higher plasma insulin concentration in our study might have contributed to the reduced concentration of plasma NEFA due to a propionate and insulin inhibitory effect on the NEFA release by adipose tissue [43].

Data from this research showed that cows fed the W20 diet had the greatest concentrations of plasma glucose and insulin. This result can be explained by the enhanced production of ruminal propionate as well as the conversion to the end product in the liver through the process of hepatic glucogenesis [44]. This finding is in accordance with a previous report from Ametaj *et al.*[8], who suggested that feeding diets containing large amounts of fermentable carbohydrates is related to a greater concentration of plasma glucose in dairy cows. Ruminal propionate and plasma glucose are secretagogues for pancreatic release of insulin, and insulin is reported to inhibit hepatic gluconeogenesis [45]. Thus, the increased plasma insulin concentration in cows fed the W20 diet, in the present study, is due to high concentration of ruminal propionate and plasma glucose associated with high FGW induced SARA.

### Oxidative stress parameters

Oxidative stress is a contributory factor to increase the susceptibility of the disease in dairy cows [46]. Lipid peroxidation (MDA) in plasma is one of the important consequences of oxidative stress, and it can be used as a reliable marker for the evaluation of the severity of oxidative stress [47]. TAC is an integrated parameter that aims to describe the dynamic equilibrium between pro-oxidants and antioxidants in the plasma compartment [48]. Here, cows fed the W20 diet had increased concentrations of MDA, and reduced levels of TAC. It is suggested that cows fed a high fermentable carbohydrate diet were more susceptible to oxidative stress than the other dietary treatments. The results were similar to a report from Hou *et al.*[49], who demonstrated that cows fed high grain diets had a lower content of serum TAC, and higher contents of serum MDA; the homeostasis of rumen and oxidative stress of the body were significantly affected by dietary F:C. Gabai *et al.*[50] reported that high-starch diets lead to an increase in oxidative stress of early lactation dairy cows, which could be caused by changes in oxidative phosphorylation. Taking into account the plasma metabolic changes (e.g., BHBA, cholesterol, and NEFA) mentioned above, the contents of MDA and TAC in plasma could be considered a reflection of the homeorhetic balance that occurs during SARA. Moreover, diets rich in readily available carbohydrates adversely affect rumen metabolism, which is associated with an increase in the yield of harmful and toxic substances (e.g., lactate, ethanol, histamine, tyramine, and endotoxin) in the rumen [51], and potentially causes systemic immune suppression and metabolic changes in dairy cows [1]. Therefore, there is a large possibility that oxidative stress plays a major role in the inflammation and immune dysfunction that occurs after induced SARA in dairy cows. The higher concentration of plasma TAC and lower concentration of plasma MDA in the cows fed the BP10 diet than the cows fed the W20 diet could be related to the higher ruminal pH than the induced SARA, and thus would decrease the negative effects of a more intensive fermentation of easily fermentable carbohydrates in the rumen.

SOD is involved in the conversion of oxygen radicals to peroxides, and GSH-Px is involved in removing the peroxides produced by SOD enzyme and converting them into water [52]. Here, a higher concentration of plasma MDA and lower concentration of plasma TAC in cows fed the W20 diet resulted in greater SOD and GSH-Px activity in plasma. It might be necessary for both enzymes to be active for adequate removal of end products of lipid peroxidation (e.g., MDA), to alleviate some of the toxic effects of reactive oxygen species (ROS), and to increase the antioxidant status of the animal [53]. A negative relationship between the activity of plasma SOD and GSH-Px and the production of plasma MDA in early lactation dairy cows was also found by Sharma *et al.*[54].

## Conclusions

SARA induced by high FGW diets resulted in strong modifications of fibre digestion, plasma metabolites, and oxidative status of dairy cows. The reduction of fibre digestion, the alterations of concentrations of selected metabolites related to carbohydrate and lipid metabolism (e.g., cholesterol, BHBA, NEFA, and triglyceride), and the changes of oxidative stress parameters (e.g., MDA, TAC, and SOD) in the plasma, are alternative candidates for diagnosis of SARA in cows of the same physiological state and environment. Substitution of pelleted BP for ground corn could reduce the risk of SARA, increase fibre digestion, and improve antioxidant status in dairy cows.

## Competing interests

The authors declare that they have no competing interests.

## Authors’ contributions

YG, XX, YZ, and ZY carried out the experiments. SL and ZC participated in the design of the study, performed the statistical analysis, and drafted the manuscript. All authors read and approved the final manuscript.
